# Specific tumor-stroma interactions of EBV-positive Burkitt's lymphoma cells in the chick chorioallantoic membrane

**DOI:** 10.1186/2045-824X-4-3

**Published:** 2012-03-09

**Authors:** Jürgen Becker, Ana Covelo-Fernandez, Frederike von Bonin, Dieter Kube, Jörg Wilting

**Affiliations:** 1 Department of Anatomy and Cell Biology, University Medicine Goettingen, 37075 Goettingen, Germany; 2 Department of Hematology and Oncology, University Medicine Goettingen, 37075 Goettingen, Germany

**Keywords:** Burkitt's lymphoma, EBV, BL2, BL2B95, BL74, Lymphatics, Dissemination, VEGF-A, VEGF-C, esVEGFR-2

## Abstract

**Background:**

Burkitt's lymphoma (BL) is an aggressive Non-Hodgkin lymphoma. Epstein-Barr Virus (EBV) is able to transform B cells and is a causative infectious agent in BL. The precise role of EBV in lymphoma progression is still unclear. Most investigations have concentrated on cell autonomous functions of EBV in B cells. Functions of the local environment in BL progression have rarely been studied, mainly due to the lack of appropriate in vivo models. Therefore, we inoculated different human BL cell-lines onto the chorioallantoic membrane (CAM) of embryonic day 10 (ED10) chick embryos and re-incubated until ED14 and ED17.

**Results:**

All cell-lines formed solid tumors. However, we observed strong differences in the behavior of EBV^+ ^and EBV^- ^cell-lines. Tumor borders of EBV^+ ^cells were very fuzzy and numerous cells migrated into the CAM. In EBV^- ^tumors, the borders were much better defined. In contrast to EBV^- ^cells, the EBV^+ ^cells induced massive immigration of chick leukocytes at the tumor borders and the development of granulation tissue with large numbers of blood vessels and lymphatics, although the expression of pro- and anti-angiogenic forms of Vascular Endothelial Growth Factors/receptors was the same in all BL cell-lines tested. The EBV^+ ^cell-lines massively disseminated via the lymphatics and completely occluded them.

**Conclusions:**

Our data suggest that the EBV^+ ^cells attract pro-angiogenic leukocytes, which then induce secondary tumor-stroma interactions contributing to the progression of BL. We show that the CAM is a highly suitable in vivo model to study the differential behavior of BL cell-lines.

## Background

With conventional chemotherapy, long-term remission can be achieved in approximately 60% of patients with disseminated "aggressive" Non-Hodgkin lymphoma (NHL) [1]. The disease incidence is increasing, but etiologic factors contributing to this phenomenon remain still largely unknown. Although it is a curable disease, many patients do not achieve complete remission, or they relapse after conventional chemotherapy. Tumor- and host-related parameters are likely to reflect some underlying biologic mechanisms and differences in the response to therapy [2,3]. One suggestion is that deregulated components of the immune system may be linked to the incidence and clinical course of lymphomas, and the development of acute or chronic inflammatory reactions at the tumor site. Cytokines, as major mediators of inflammation, were found to be associated with the transformation of lymphatic malignancies either as autocrine growth factors for the transformed cells or as factors rebuilding the tumor microenvironment, likely affecting tumor progression and dissemination.

More than 50 years ago, Dennis Burkitt (1958) described the high incidence of a very aggressive lymphoma in young children in equatorial Africa, which now belongs to the group of NHL [4]. Later on by Anthony Epstein, a herpes virus was identified in these lymphoma cells. The Epstein-Barr virus (EBV), which is found in appr. 95% of Burkitt's lymphoma (BL) cases in subsaharean Africa, contributes to the pathogenesis of BL, obviously in conjunction with a chronic severe and thus most probably immunosuppressive *Plasmodium falciparum *malaria infection [5,6]. However, numerous questions about the effects of EBV in BL have remained unanswered [7]. This is partially due to the lack of appropriate experimental in vivo models to study the progression of the disease. In vivo models are of utmost importance, because lymphoma progression can be controlled by interactions with the local environment, mostly mediated by cytokines, chemokines, morphogens and/or growth factors [8].

Despite their different cellular origins and divers molecular signatures, lymphomas posses similar routes for dissemination. Patients initially present with lymph node swelling or primary lesions in lymphatic organs. Dissemination takes place via lymphatic tissues and tumor cells thereby reach downstream lymph nodes as well as extra-lymphatic tissues. The clinical staging takes into account the number of involved lymph nodes, below and above the diaphragm. In highly progressed stages, disseminated lymphoma cells are found in the central nervous system, liver and the peritoneal cavity, and obviously these organs become infiltrated by hematogenic dissemination - although direct connections (stomata) between the peritoneal cavity and the lymphatics might as well serve as a route for lymphogenic dissemination [9,10].

There are indications that lymphoma dissemination reflects the physiological migratory behavior of lymphocytes along blood and lymphatic vessels, and is not necessarily a sign for tumor progression [11]. However, high vascularity was found in the bone marrow of children with acute lymphoblastic leukemia and a positive correlation between the hemangiogenic protein Vascular Endothelial Growth Factor-A (VEGF-A) and lymphoma progression has been observed [12,13]. Lymphangiogenic growth factors, most importantly VEGF-C and VEGF-D [14-16], increase the number of lymph node metastases of numerous carcinomas [17]. Thereby, proangiogenic growth factors can be counteracted by endogenous inhibitors. These are membrane-bound and soluble forms of VEGFR-1 to inhibit VEGF-A, and the endogenous soluble monomeric form of VEGFR-2 (esVEGFR-2), which inhibits VEGF-C [17-19]. The ligands and their membrane-bound receptors have been studied in various types of lymphomas [20-25]. However, the endogenous soluble inhibitors have not been studied yet, although they seem to control the progression of childhood tumors such as neuroblastoma [26]. The effects of EBV on the expression of pro- and anti-hem/lymphangiogenic members of the VEGF family, and the local patterns of EBV^+ ^vs. EBV^- ^lymphoma cell dissemination have not been studied in detail. We therefore compared the migratory behavior of EBV^+ ^and EBV^- ^human BL cell-lines and their interactions with the stroma in xenografts on the chick chorioallantoic membrane (CAM). The CAM is densely supplied with both blood and lymphatic vessels [27] and well suited to study tumor-host interactions [28]. Furthermore we used real-time RT-PCR to quantify the expression of VEGFs and their receptors in BL cell-lines. We observed significant differences in the behavior of EBV^+ ^and EBV^- ^BL cell-lines, although the expression of VEGFs did not differ between the cell-lines.

## Results

**Figure 1:**
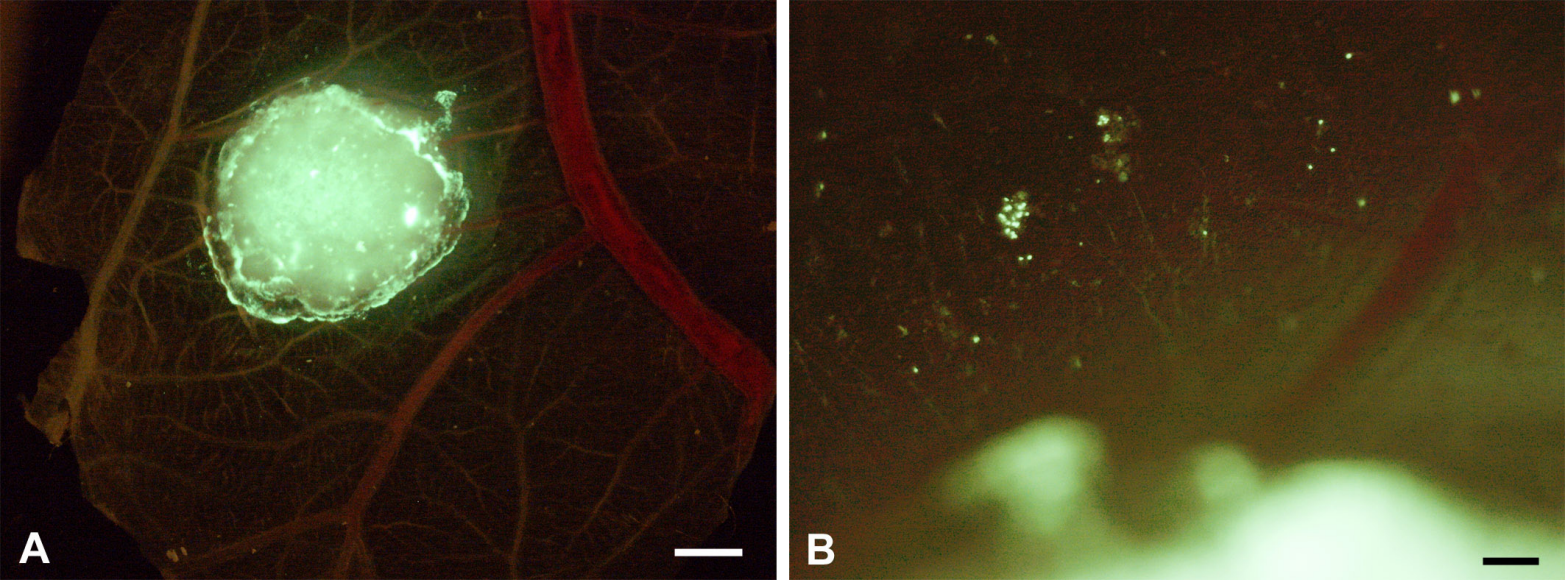
**BL2 cells stained with cell-tracker (green) and incubated on the CAM for 4 days**. **A) **The cells form a solid tumor with almost sharp borders. CAM vessels are faintly visible. Bar = 2 mm. **B) **Higher magnification of A) showing a few green tumor cells at some distance from the solid tumor. Bar = 150 μm.

**Figure 2:**
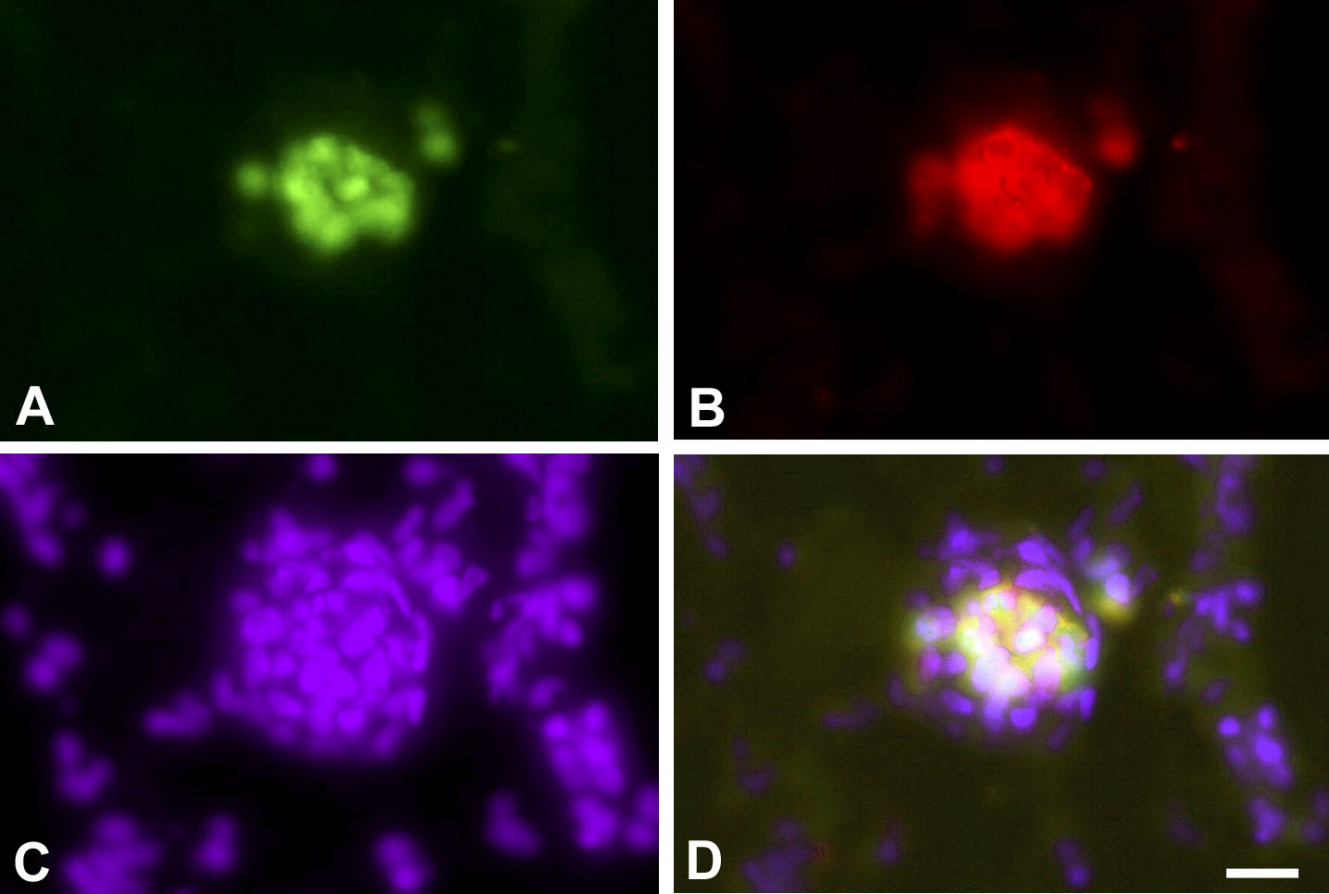
**Staining of metastatic BL2 cells in the CAM**. **A) **BL2 cells are marked with cell-tracker green. **B) **BL2 cells stain with anti-human-HLA antibodies (red). **C) **Staining of all nuclei with Dapi (blue). **D) **Merged picture. Bar = 60 μm.

We incubated three human Burkitt lymphoma (BL) cell-lines on the chick chorioallantoic membrane (CAM). We chose the EBV^- ^cell-line BL2, and the EBV^+ ^cell-lines BL2B95 and BL74. Inoculation of cells was on day 10 of chick embryo development, and tumors were studied after 4 or 7 days. The EBV^- ^BL2 cells formed solid tumors with relatively sharp borders (Figure 1A). Only a small number of lymphoma cells were seen in the CAM at a distance of several hundred micrometers from the tumor mass (Figure 1B). To verify that these cells were lymphoma cells that had invaded the stroma of the CAM (and not macrophages that had taken up the cell tracker by engulfing tumor cells), we stained the specimens with anti-human HLA antibodies. There was a clear overlap of the HLA signal (red) with the cell-tracker (green) (Figure 2), showing that tumor cells had invaded the CAM stroma.

**Figure 3:**
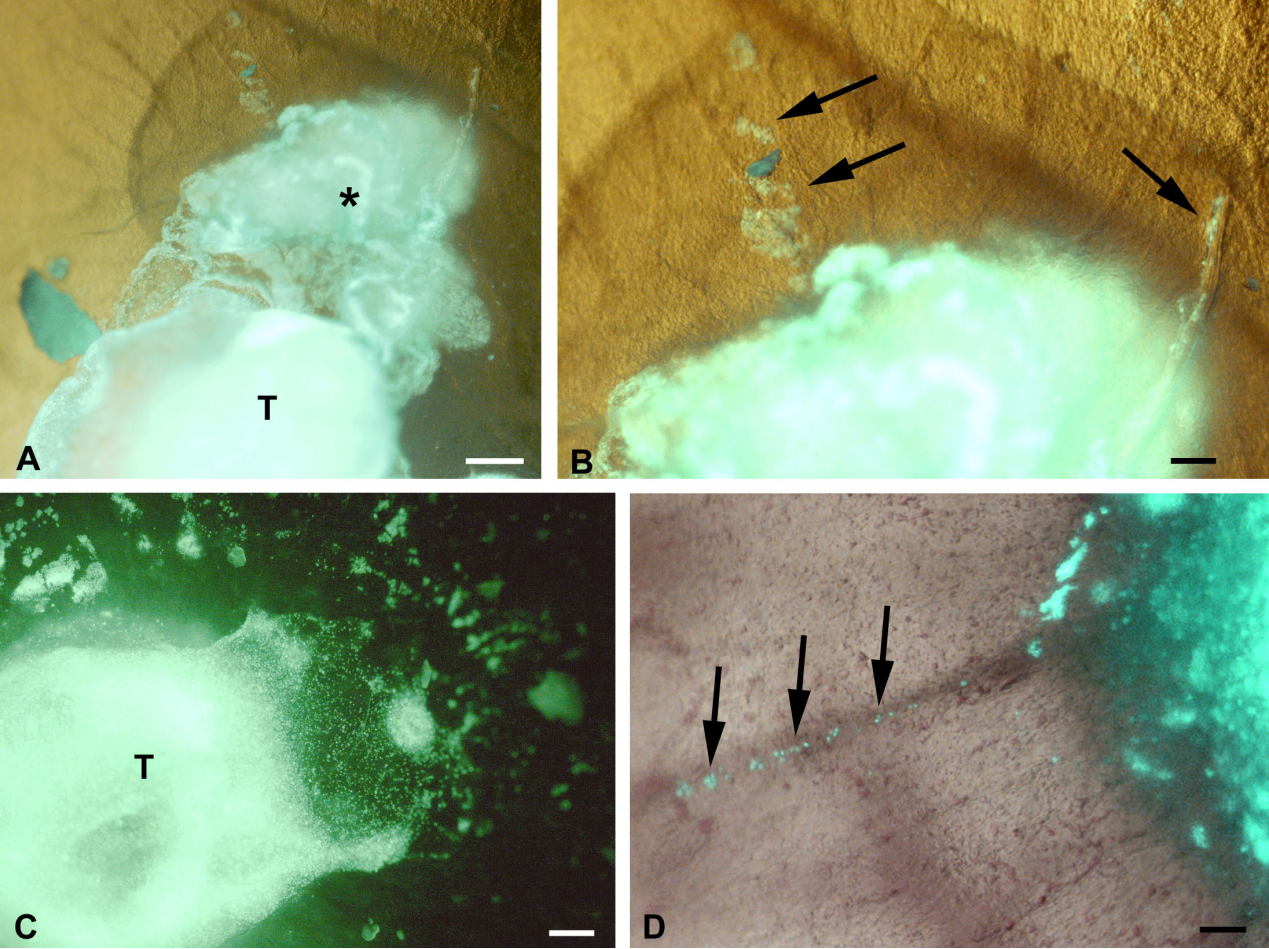
**BL2B95 cells stained with cell-tracker (green) and incubated on the CAM for 4 days**. The cells form solid tumors. The tumor borders are less sharp than those of BL2 cells. CAM vessels are faintly visible. **A) **A great mass of tumor cells (asterisk) is seen adjacent to the solid tumor (T). Bar = 2,5 mm **B) **Higher magnification of A). Note tumor cells (arrows) that obviously migrate from the tumor. Bar = 150 μm. **C) **Numerous scattered lymphoma cells and clusters of lymphoma cells are present at some distance from the solid tumor (T). Bar = 300 μm. **D) **Note lymphoma cells (arrows) lining up along a CAM vessel. Bar = 150 μm.

**Figure 4:**
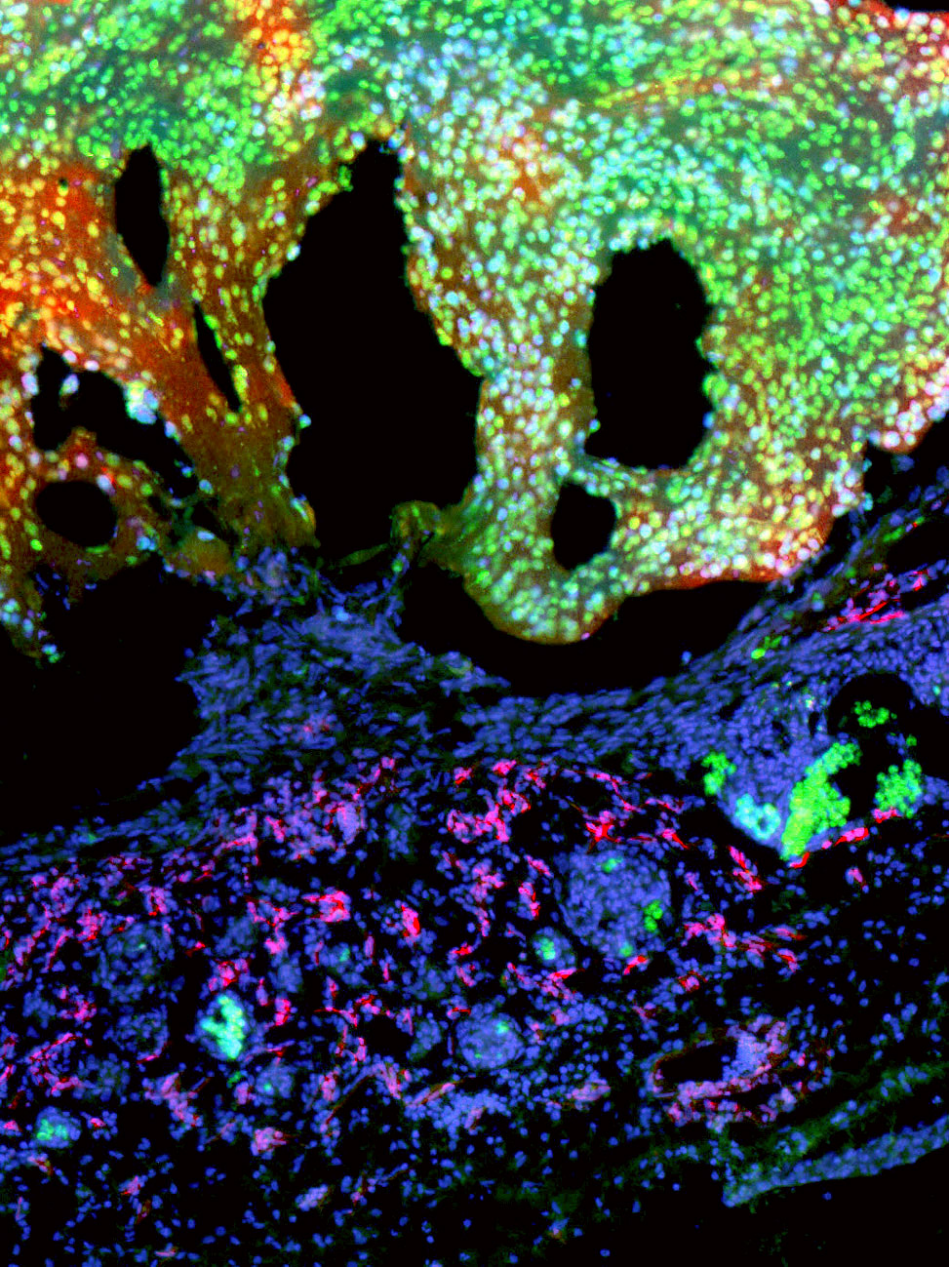
**BL2B95 cells stained with cell-tracker (green) form solid tumors (T) on the CAM after 4 days**. Clusters of lymphoma cells (arrows) have invaded the CAM. Mep 21 staining (red) reveals a large amount of blood capillaries in the CAM. Bar = 100 μm.

The EBV^+ ^BL2B95 and BL74 cells showed a somewhat different behavior. The borders of the tumors were much more irregular and frayed (Figure 3A-D). Many scattered cells were present at the tumor borders (Figure 3C). In most specimens we observed rows of tumor cells that obviously followed preformed pathways into the CAM (Figure 3B,D). In immunohistological specimens we observed clusters of lymphoma cells in the CAM stroma, and high densities of blood capillaries adjacent to the tumors (Figure 4).

**Figure 5:**
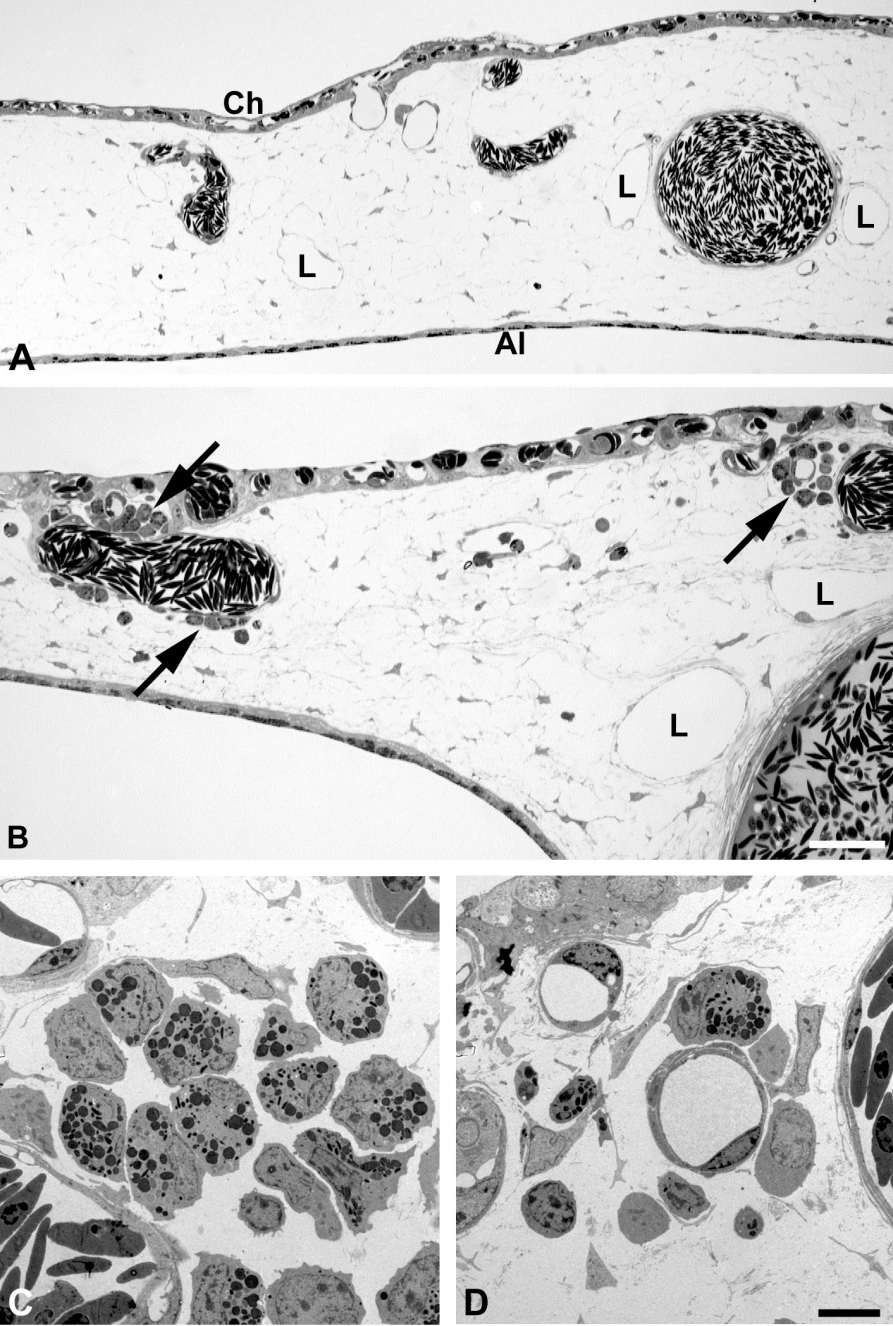
**Semi- and ultrathin sections of CAM tissue approximately 7 mm apart from the solid tumor**. **A) **In BL2 specimens the typical morphology of the CAM is found with capillaries located in the chorionic epithelium (Ch), larger blood vessels and lymphatics (L) in the stroma, and the allantoic epithelium (Al). Bar = 80 μm. **B) **In BL2B95 specimens large numbers of leukocytes (arrows) are found in perivascular position. **C, D) **Ultra-thin sections of the specimen shown in B). Note invasion of granulocytes and other leukocytes into the CAM stroma. Bar = 10 μm.

**Figure 6:**
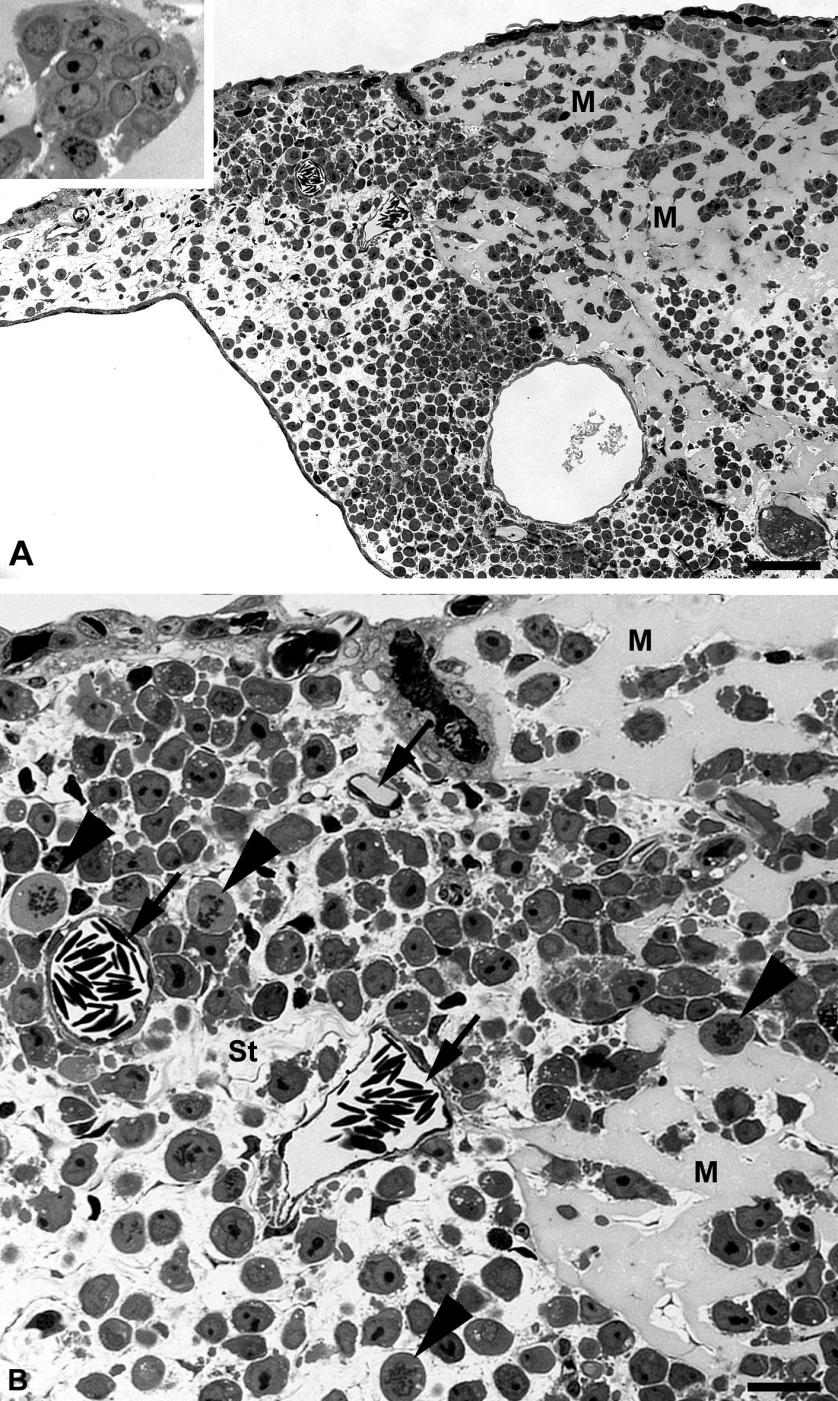
**Semi-thin sections of BL2 tumors**. **A) **Tumor cells were applied on the CAM within Matrigel, which is still visible after 7 days as amorphous material (M). Ch, chorionic epithelium; Al, allantoic epithelium. Insert: Higher magnification of lymphoma cells in Matrigel. Note the large nuclei with one or several nucleoli. Bar = 120 μm. **B) **Higher magnification of A) showing tumor cells in Matrigel (M). Tumor cells have invaded the CAM stroma (St). Blood vessels (arrows); Mitotic figures of lymphoma cells (arrowheads). Bar = 30 μm.

**Figure 7:**
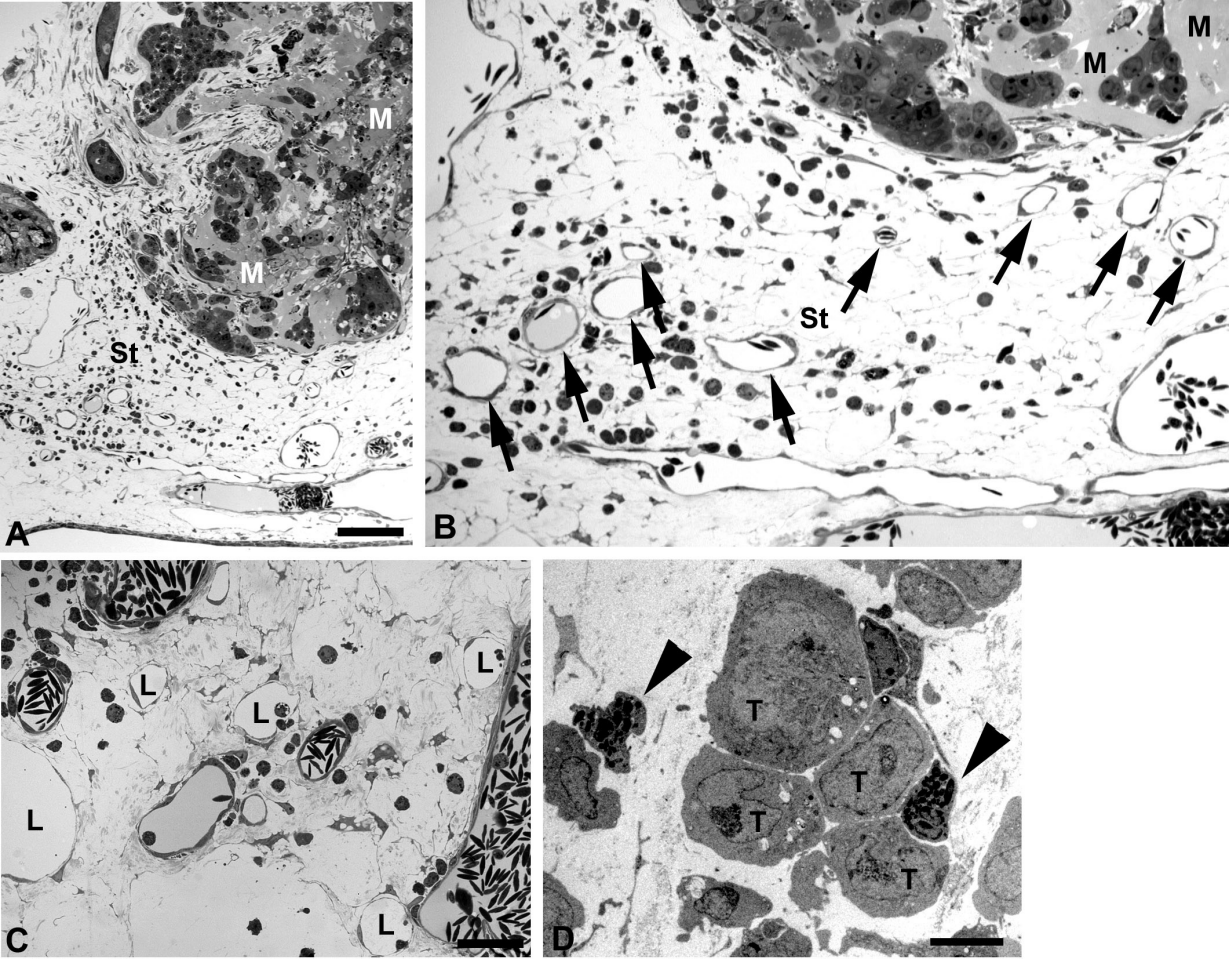
**Semi-thin sections of BL74 and BL2B95 tumors**. **A) **BL74 cells were applied on the CAM within Matrigel (M), which is visible after 4 days as amorphous material. Al, allantoic epithelium. Note the granulation tissue in the CAM stroma (St). Bar = 120 μm **B) **Higher magnification of A) showing tumor cells in Matrigel (M). Note the large number of leukocytes (small round cells) and the numerous blood vessels (arrows) in the CAM stroma. **C) **Peritumoral stroma of a BL2B95 tumor. Note large numbers of blood vessels and lymphatics (L). Bar = 30 μm in B,C. **D) **Ultra-thin section showing tumor cells (T) in the CAM stroma immediately accompanied by chick leukocytes (arrowheads). Bar = 15 μm.

In semi-and ultrathin sections we observed distinct differences in the behavior between the EBV^+ ^and EBV^-^cell lines. We started semi-thin sectioning at a distance of approximately 7 mm from the lymphoma. In BL2 specimens there was the regular morphology of the CAM with chorionic and allantoic epithelium, stroma, blood and lymphatic vessels (Figure 5A). In BL2B95 and BL74 specimens, large numbers of perivascular leukocytes were found (Figure 5B-D). These cells obviously invaded the stroma via postcapillary venules. This difference in leukocyte numbers was consistently seen in EBV^+ ^vs. EBV^- ^cases. Furthermore, for BL2 we found lymphoma cells that were still embedded in Matrigel, as well as cells that had invaded the adjacent stroma (Figure 6A,B). The number of peritumoral blood vessels was low and these vessels were seemingly only the pre-existing ones (Figure 6A,B). In contrast, for BL2B95 and BL74 we found large numbers of both leukocytes as well as blood and lymphatic vessels in the peritumoral stroma (Figure 7A-C). Lymphoma cells that invaded the stroma of the CAM were in immediate contact with the leukocytes (Figure 7D). The results are summarized in Table 1.

**Table 1:** Summary of results obtained with EBV^+ ^and EBV^- ^BL cell-lines.

Cell-line	EBV	Tumor borders	Host's leukocyte infiltration	Hem-angiogenesis	Lymph-angiogenesis	Lymphogen. dissemination	VEGF-A	VEGF-C
BL2	-	rel.sharp	low	-	-	+	-	-

BL2B95	+	Frayed	high	+	+	+++	-	-

BL74	+	frayed	high	+	+	+++	n.d.	n.d.

Positve (+) and negative (-) results are marked; n.d. = not determined

**Figure 8:**
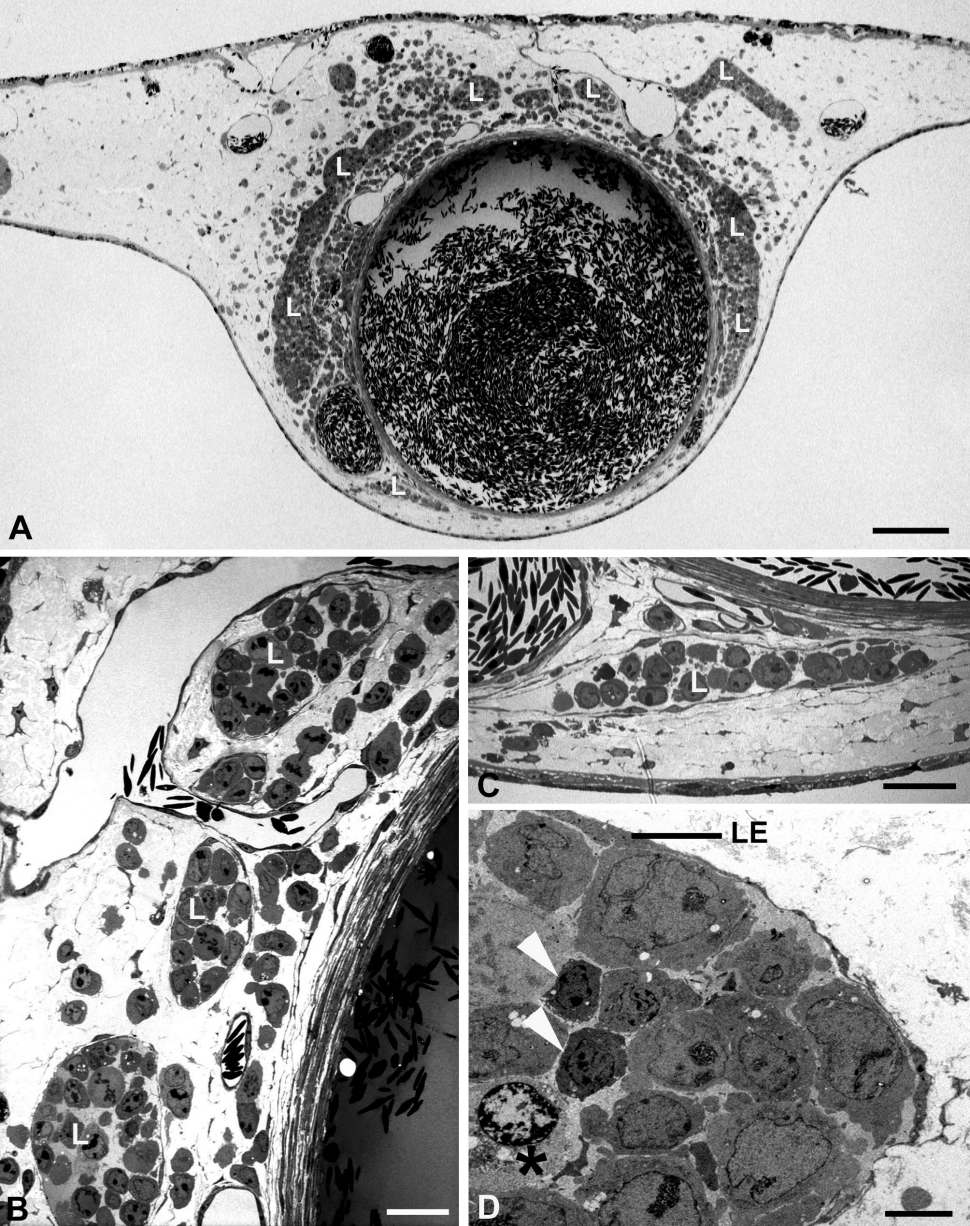
**Semi- and ultra-thin sections of BL2B95 tumors**. **A) **At approximately 5 mm distance from the solid tumor, some lymphoma cells are found in the CAM stroma. The CAM lymphatics (L) are completely filled with tumor cells. Bar = 300 μm. **B - D) **Higher magnification of the specimen in A). **B, C) **Semi-thin sections. The lymphatics (L) are completely filled with tumor cells. Bar = 30 μm in B, and 40 μm in C. **D) **Ultra-thin section showing lymphatic endothelium (LE) and tumor cells, which fill the vessel completely. Two leukocytes (arrowheads) are visible. One is in contact with a dying lymphoma cell (asterisk), as seen by the heterochromatin condensation in the periphery of the nucleus. Bar = 10 μm.

**Figure 9:**
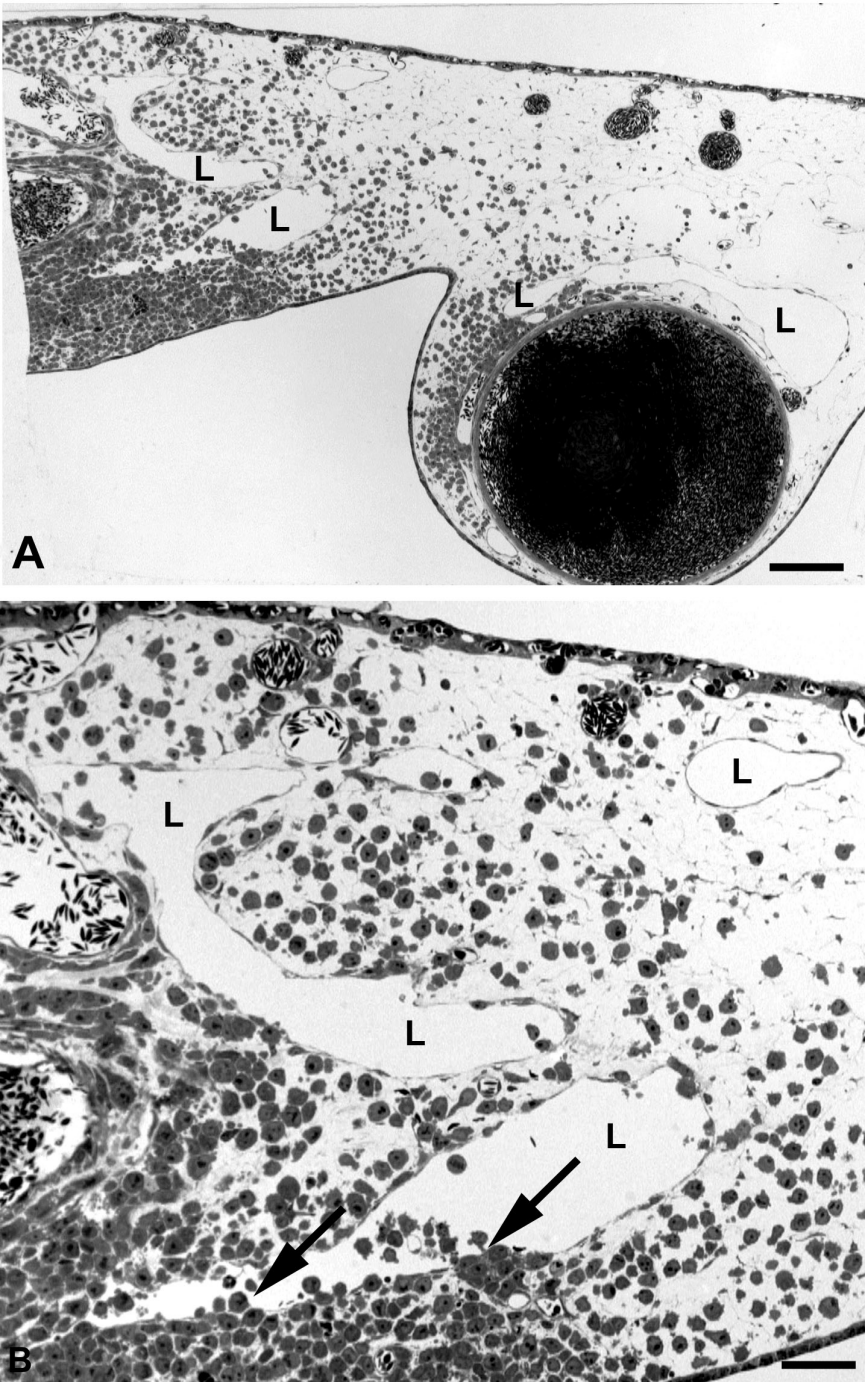
**Semi-thin sections of BL2 tumors**. **A) **Immediately adjacent to the solid tumor (on the left side of the specimen), numerous lymphoma cells have invaded the CAM stroma. Only very few cells are found in the CAM lymphatics (L). Bar = 400 μm. **B) **Higher magnification of A) showing CAM lymphatics (L) invaded by only a few tumor cells (arrows). Bar = 80 μm.

Besides the large numbers of leukocytes and peritumoral vessels, we observed another significant difference between EBV^+ ^and EBV^- ^cell-lines. BL2B95 and BL74 cells invaded lymphatic vessels at a much higher rate as compared to BL2 cells. Although all cell-lines possessed the potency to invade both blood an lymphatic vessels, it became immediately obvious that the EBV^+ ^cells completely filled the lymphatics, even at large distances from the solid tumors (Figure 8A-D). In contrast, the BL2 cells migrated within the stroma, and rarely invaded the lymphatic vessels (Figure 9A,B).

In summary, the morphological studies showed that the EBV^+ ^cell-lines BL2B95 and BL74 induced a stronger immigration of chick leukocytes into the stroma at the tumor borders, more blood and lymphatic vessels, and they invaded the lymphatics much more strongly than the EBV^- ^BL2 cells (Table 1).

**Figure 10:**
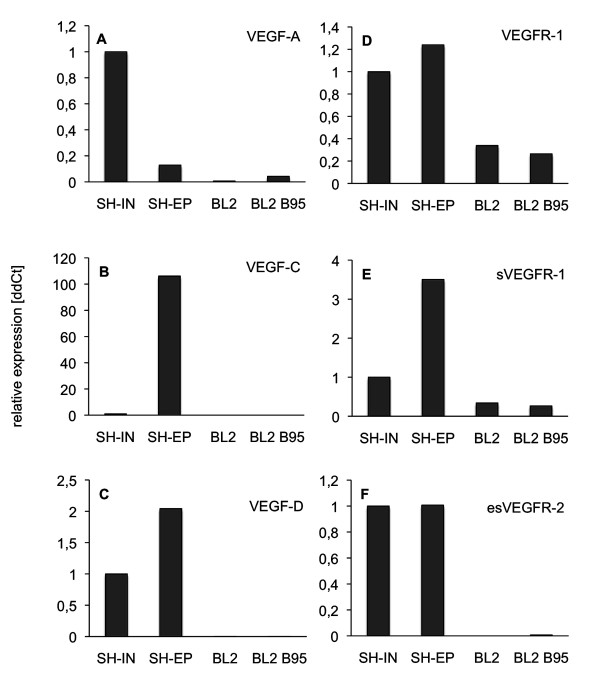
**Real-time RT-PCR of BL2 and BL2B95 lymphoma cells in comparison with neuroblastoma (NB) cells**. Expression of **A) **VEGF-A, **B) **VEGF-C, **C) **VEGF-D, **D) **VEGFR-1, **E) **sVEGFR-1 and **F) **esVEGFR-2. As compared to NB cells Shep and SK-IN there is no significant expression of the growth factors in the BL2 and BL2B95 cells. Membrane-bound and soluble forms of VEGFR-1 are weakly and equally expressed in the lymphoma cell-lines, while esVEGFR-2 is not expressed.

It has frequently been observed that hematogenic and lymphogenic dissemination of tumor cells correlates with the expression of hem-and lymphangiogenic growth factors, especially those of the Vascular Endothelial Growth Factor (VEGF) family. Therefore, we quantified by real-time RT-PCR the expression of VEGF ligands and VEGF receptors in BL2 and BL2B95 cells in comparison with the neuroblastoma cell-lines SH-IN and SH-EP. The expression of VEGF-A in the two BL cell-lines was negligible (Figure 10A). We did not find any expression of the lymphangiogenic factors VEGF-C and VEGF-D in BL2 and BL2B95 (Figure 10B,C; Table 1). The membrane-bound form of VEGFR-1 and the soluble form of the receptor, sVEGFR-1, were weakly and equally expressed in the BL2 and BL2B95 cells (Figure 10D,E). We did not observe any expression of the membrane-bound VEGFR-2 (data not shown) and secreted esVEGFR-2 in the two BL cell-lines (Figure 10F). Therefore, we hypothesize that the distinct tumor-vessel interactions of the BL cells are not regulated directly by VEGFs produced in the tumor cells, but indirectly by the attracted leukocytes.

## Discussion

It is known for long that the chorioallantoic membrane (CAM) of chicken embryos can be used for tumor engraftment [29], since the adaptive immune system is not mature until the third week after hatching [30]. However, all types of blood cells and leukocytes develop already *in ovo*. Firstly, erythrocytes (embryonic day 2, ED2) and platelets (ED4) are found in the blood. Myelopoietic precursor cells (ED6) originate from the yolk sac, and from ED8 onwards, erythropoietic and granulopoietic cell islands are found in the liver [31,32]. CD3-positive pre-T-cells are found in the thymus on ED9 and T cell receptor-1 and -2 are expressed on ED12 [33]. First indications of B cell development can be seen on ED6 and migration into the bursa of Fabricius takes place on ED10 [34,35]. We inoculated the human lymphoma cell-lines BL2, BL2B95 and BL74 in Matrigel on the CAM of ED10 chick embryos and re-incubated until ED14 and ED17. During this period, all types of leukocytes are present in the embryo.

In this study we observed, for the first time in an in vivo model, different reactions of the host tissue to EBV^+ ^BL cells as compared to EBV^- ^BL cells. The EBV^+ ^cell-lines induced massive immigration of chick leukocytes into the stroma at the tumor borders, more blood and lymphatic vessels, and they invaded and completely filled the lymphatics even at large distances from the solid tumor. After 4 days of re-incubation, the borders of the EBV^+ ^tumors were much more irregular and numerous tumor cells were seen invading the CAM. In contrast, the borders of the EBV^- ^tumors were much better defined. We did not observe any differences in the expression of the hem- and lymphangiogenic growth factors VEGF-A, -C and -D, or their inhibitors VEGFR-1, sVEGFR-1 and esVEGFR-2. In fact, the RNA expression levels for VEGF-A and -C were extremely low, or absent, in BL2 and BL2B95, as compared to the NB cell-lines SH-IN and SH-EP. In the latter cell-lines we have previously quantified protein levels and found: VEGF-A = 192 pg/ml supernatant for SH-EP and 2005 pg/ml for SH-IN; and VEGF-C = 140 pg/ml for SH-EP and below detection level for SH-IN [26]. RNA and protein levels corresponded very well. Therefore, it is very likely that no significant VEGF-A and -C protein levels are detectable in BL2 and BL2B95. We therefore assume that the differential tumor-vessel interactions of the EBV^+ ^cell-lines BL2B95 and BL74 as compared to the EBV^- ^cell-line BL2 are due to the immigration of leukocytes induced by the former. The production of angiogenic growth factors and matrix metalloproteinases by immune cells is well documented [36].

EBV is one out of eight members of the gamma herpes-virus family, which have co-evolved with man. It has established a mostly harmless but complex co-existence in B cells. Life-long EBV infection is found in approximately 95% of the world's population [37]. EBV is the causative agent in infectious mononucleosis. However, its role in carcinogenesis is still poorly understood. EBV seems to contribute to cellular transformation in a defined window of B cell differentiation, but transformed cells are usually recognized by the immune system [38]. EBV is capable of transforming various cell types. Most studies on EBV in BL have concentrated on direct functions of the viral genome in the infected cells. However, lymphoma progression can also be controlled by interactions with the local environment [8]. We observed specific interactions of the EBV^+ ^BL cells with the chick lymphatics. These were completely filled with tumor cells, even at large distances from the solid tumor. The EBV^- ^BL2 cells migrated through the stroma and only few of them invaded the lymphatics. Lymph node involvement is an important criterion for clinical staging of lymphomas. A direct involvement of the lymphatics has been observed in the rare cases of BL in immunocompetent patients, where the disease is diagnosed in the orbita [39]. These patients show eyelid edema and systemic involvement of the lymphatics. In numerous cancers, the degree of lymphogenic metastases is positively correlated with the expression of the pro-lymphangiogenic factors VEGF-C and -D [17,40]. Here, we did not find any differences in the expression of pro- and anti-lymphangiogenic factors in the three BL cell-lines. However, we observed massive infiltration of leukocytes into EBV^+ ^tumors, and higher vascular density. VEGF-C is expressed in macrophages, dendritic cells and neutrophils, and up-regulated by the pro-inflammatory cytokines IL-1α, IL-1β and tumor necrosis factor-α [41]. We therefore propose that specific virus-host interactions and secondary tumor-stroma interactions contribute to the progression of BL via the lymphatic vascular system.

## Conclusions

The mechanisms by which EBV contributes to the progression of BL are poorly understood. The increased resistance to apoptosis of infected cells has been shown in many studies. Modulation of the micro-environment has also been regarded as an important aspect, but has been difficult to study due to the paucity of in vivo models. Our studies on EBV^+ ^and EBV^-^BL cell-lines in the chick chorioallantoic membrane seem to support the concept of virus-mediated interactions with the vascularized stroma. Although the tested cell-lines have almost identical VEGF and VEGF-receptor expression profiles they interact differentially with the vascular system, both the blood vessels and the lymphatics. Thereby, EBV^+ ^BL cells induce massive immigration of leukocytes, which may then induce hem- and lymphangiogenesis. It is likely that the latter promotes lymphogenic dissemination of tumor cells.

## Materials and methods

### Cell culture

The human BL cell lines BL74 and BL2B95, which are EBV^+ ^[42], the BL2 cells, which are EBV^- ^[43], and the human neuroblastoma cell-lines SH-IN and SH-EP [26] were maintained in a humidified incubator at 37°C and 5% CO_2 _atmosphere using RPMI 1640 medium (Lonza, Basel, Switzerland) with 10% fetal bovine serum (Biochrome, Berlin, Germany) and 1% penicillin/streptomycin (Invitrogen, Darmstadt, Germany).

### Labeling of cells

For the in vivo labeling of BL cells we used cell tracker-green (CFDA; Molecular probes) at a final concentration of 6 μM in serum-free medium for 45 min at 37°C. Then, cells were incubated for 30 min in RPMI with 10% serum and 1% pen/strep. Cells were washed in phosphate buffered saline and mixed with Matrigel:serum (1:1) (B&D, Heidelberg, Germany).

### Chick chorioallantoic membrane (CAM) assay

Fertilized White Leghorn chick eggs were incubated at 37.8°C and 80% relative humidity. A window was made into the egg shell at day three and sealed with cellotape. Eggs were placed back in the incubator and at day 10 the lymphoma cells (1 × 10^6 ^in Matrigel/serum; B&D) were applied on the CAM. Tumors were studied at day 14 and day 17 of chick development. Experimental BL2 tumors (n = 16), BL2B95 tumors (n = 18) and BL74 tumors (n = 8) were studied.

### Semi- and ultra-thin sectioning

Specimens were fixed in Karnovsky's fixative, post-fixed in osmium tetroxide solution and embedded in Epon resin (Serva, Heidelberg, Germany) according to standard techniques. Semi-thin sections of 750 nm were stained with Richardson solution and studied with a light microscope. Ultra-thin sections of 70 nm were contrasted with lead citrate and uranyl acetate and studied with a transmission electron microscope (TEM) (Zeiss, Göttingen, Germany).

### Immunohistology

Specimens were fixed for 15-20 min. with 4% paraformaldehyde (PFA), rinsed three times in PBS, transferred into 10% and 30% saccarose, and embedded in tissue freeze medium (Sakura Finetek Europe, NL). Primary antibodies were HLA (Becton Dickinson) and Mep21 (chick CD34 homolog; M. Williams, AbLab, University of British Columbia, Vancouver, B.C., Canada; also see: [44]). Secondary antibodies were Alexa 594-conjugated goat-anti-mouse IgG (Molecular Probes, Eugene, USA), applied at 1:200 dilution. Sections were studied with Zeiss Axio Imager Z1 (Zeiss, Göttingen, Germany).

### Real-time RT-PCR

We prepared cDNA from 2 μg total RNA with Omniscript reverse transcriptase (Qiagen, Hilden, Germany). Real-time RT-PCR was performed with Opticon2 thermal cycler (MJ Research, Waltham, MA), using SYBR green JumpStart Taq ReadyMix (Sigma-Aldrich, Taufkirchen, Germany). For esVEGFR-2 the reverse primer recognizes the motif in intron 13, which is specific for the endogenous soluble splice-variant of VEGFR-2 [18]. The probes were normalized using β-actin probes. Relative expression levels of transcripts were calculated with the ΔΔCt-method using Microsoft Excel 2008 for Mac (Microsoft Corp. Redmond, WA). Primers are listed in Table 2.

**Table 2:** Primers used for real-time RT-PCR

Target Name	Primer Sequence
VEGF-A fwd.	5'- AAGGAGGAGGGCAGAATCAT -3'

VEGF-A rev.	5'- GCAGTAGCTGCGCTGATAGA -3'

VEGF-C fwd.	5'- TGAACACCAGCACGAGCTAC -3'

VEGF-C rev.	5'- GCCTTGAGAGAGAGGCACTG -3'

VEGF-D fwd.	5'- TGGAACAGAAGACCACTCTCATCT -3'

VEGF-D rev.	5'- GCAACGATCTTCGTCAAACATC-3'

VEGFR-1 fwd.	5'- TCCAAGAAGTGACACCGAGA -3'

VEGFR-1 rev.	5'- TTGTGGGCTAGGAAACAAGG -3'

sVEGFR-1 fwd.	5'- GCACGTTTGGATTTGGAGGA -3' [45]

sVEGFR-1 rev.	5'- GGAAAGGATCATCCCAAGTTGTT -3' [45]

esVEGFR-2 fwd.	5'- GCCTTGCTCAAGACAGGAAG -3'

esVEGFR-2 rev.	5'- CAACTGCCTCTGCACAATGA -3'

β-actin fwd.	5'- GCATCCCCCAAAGTTCACAA -3'

β-actin rev.	5'- AGGACTGGGCCATTCTCCTT -3'

## Competing interests

The authors declare that they have no competing interests.

## Authors' contributions

JB designed and established experiments, designed primers, performed real-time RT-PCR experiments and worked on the manuscript. AC-F performed CAM experiments and performed and analyzed immunohistology. FvB did Lymphoma cell culture. DK provided cell lines, designed experiments and prepared the manuscript. JW designed experiments, analyzed data and prepared the manuscript. All authors read and approved the final manuscript.
